# Supplementary material for: Direct Metatranscriptome RNA-seq and Multiplex RT-PCR Amplicon Sequencing on Nanopore MinION – Promising Strategies for Multiplex Identification of Viable Pathogens in Food
Source: Front Microbiol. 2020 Apr 9;11:514. doi: 10.3389/fmicb.2020.00514 (PMC7160302; doi:10.3389/fmicb.2020.00514)
Supplement: Supplementary file 1 [file Data_Sheet_1.docx]

***Supplementary Table***

**Supplementary Table 1. Summary of conventional cultivation and commercial rapid detection methods.**

| Method | Rapidness (hours)* | VBNC* | Portability* | Viability* | Multiplex w/o customization* |
| --- | --- | --- | --- | --- | --- |
| Most probable number | **24-48** | **No** | Yes | Yes | Yes |
| Viable counts (e.g., Petrifilm^TM^) | **24-72** | **No** | Yes | Yes | Yes |
| Lateral flow | <1 | **No** | Yes | **No** | **No** |
| DEFT/SPC | <1 | **No** | **No** | **No** | **No** |
| Immunoassay | 1-4 | **Enrichment required** | Yes | **Enrichment required** | **No** |
| Flow cytometry | <1 | Yes | **No** | Yes | **No** |
| MALDI-TOF | 1 | **No** | **No** | Yes | Yes |
| DNA/PCR | ~4 | Yes / **False positive** | Yes | **No** / **False positive** | **No** |
| RNA/RT-PCR | ~4 | Yes / **False positive** | Yes | Yes / **False positive** | **No** |
| Next generation sequencing (NGS) | 1-4 | Yes | **No** | Yes | Yes |
| **Nanopore sequencing** | **1-4** | **Yes** | **Yes** | **Yes** | **Yes** |

* Bold indicates major limitations.

**Supplementary Table 2. Nanopore sequencing input amount of RNA and RT-PCR amplicon (cDNA).** CFU number was measured by plating count and nucleotide concentration was tested by Qubit assay. *Ec*, *Se* and *Lm* indicated *E. coli* O157:H7, *S. enteritidis* and *L. monocytogenes*, respectively. The bacteria culture condition was 37°C incubation.

| Sequencing | Nanopore sequencing - Direct metatranscriptome RNA-seq | | | | | | RT-PCR amplicon | | | | | | Next generation sequencing - Direct metatranscriptome RNA-seq | | | | | |
| --- | --- | --- | --- | --- | --- | --- | --- | --- | --- | --- | --- | --- | --- | --- | --- | --- | --- | --- |
| Sample | BHI 336 24h | | | LJE 336 24h | | | BHI 334 4h | | | LJE 334 4h | | | BHI 336 24h | | | LJE 336 24h | | |
| Initial culture conc. (Log CFU/mL) | *Ec* | *Se* | *Lm* | *Ec* | *Se* | *Lm* | *Ec* | *Se* | *Lm* | *Ec* | *Se* | *Lm* | *Ec* | *Se* | *Lm* | *Ec* | *Se* | *Lm* |
|  | 2.9 | 3.1 | 4 | 3.5 | 3.5 | 6 | 3.6 | 3.5 | 5 | 3.6 | 3.3 | 6 | 2.3 | - | 5 | 2.9 | 3.3 | 5 |
| 24h/4h culture conc. (Log CFU/mL) | *Ec* | *Se* | *Lm* | *Ec* | *Se* | *Lm* | *Ec* | *Se* | *Lm* | *Ec* | *Se* | *Lm* | *Ec* | *Se* | *Lm* | *Ec* | *Se* | *Lm* |
|  | 8.7 | 9.2 | 7 | 8.6 | 8.0 | 5 | 5.3 | 5.0 | 6 | 4.5 | 3.6 | 5 | 9.4 | 7.7 | 9 | 5.2 | 5.2 | 6 |
| Sample size (mL) | 5.3 | | | 5.0 | | | 0.2 | | | 0.2 | | | 0.04 | | | 0.24 | | |
| RNA input for multiplex RT-PCR (ng) | - | | | - | | | 72.0 | | | 36.5 | | | - | | | - | | |
| Yield of cDNA from multiplex RT-PCR (ng) | - | | | - | | | 1267.5 | | | 705.2 | | | - | | | - | | |
| RNA/DNA input for library prep (ng) | 3490.2 | | | 1338.0 | | | 33.8 | | | 165.0 | | | 100.0 | | | 100.0 | | |
| Yield after Poly A tailing and purification (ng) | 1824.0 | | | 617.5 | | | - | | | - | | | - | | | - | | |
| Yield after library prep and sequencing input (ng) | 744.0 | | | 129.2 | | | <30.0 Too low to detect | | | 125.0 | | | <1 (1 nM) | | | <1 (1 nM) | | |

**Supplementary Table 3. Primer information for qPCR, RT-qPCR and multiplex RT-PCR.**

| **Species** | **Target Gene*** | **Primer (5'-3')** | **Product Size (bp)** | **Reference** |
| --- | --- | --- | --- | --- |
| *Ec* | *stx* | F: GAGCGAAATAATTTATATGTG | 520 | (Zhang et al., 2000; Toma et al., 2003; Nguyen et al., 2016) |
|  |  | R: TGATGATGGCAATTCAGTAT |  |  |
|  | *stx1A* | F: TGACAGGATTTGTTAACAGGAC | 294 | (Zhang et al., 2000; Toma et al., 2003; Nguyen et al., 2016) |
|  |  | R: TCTGTATTTGCCGAAAACGT |  |  |
| *Se* | *invA* | F: ACAGTGCTCGTTTACGACCTGAAT | 244 | (van der Velden et al., 2000; Nguyen et al., 2016) |
|  |  | R: AGACGACTGGTACTGATCGATAAT |  |  |
| *Lm* | *inlA* | F: GATTAACACGAGTAACGG  R: TAGATCTGTTTGCGAGAC | 153 | (Vázquez-Boland et al., 2001; Xiao et al., 2012; Nguyen et al., 2016) |

* *stx1A* was used in qPCR and RT-qPCR of *E. coli* O157:H7. *stx*, *invA* and *inlA* were used in multiplex RT-PCR of 4-hour BHI and 4-hour LJE cocktail cultures.

**REFERENCES**

Nguyen, T.T., Van Giau, V., and Vo, T.K. (2016). Multiplex PCR for simultaneous identification of *E. coli* O157:H7, *Salmonella spp*. and *L. monocytogenes* in food. *3 Biotech* 6(2)**,** 205. doi: 10.1007/s13205-016-0523-6.

Toma, C., Lu, Y., Higa, N., Nakasone, N., Chinen, I., Baschkier, A., et al. (2003). Multiplex PCR assay for identification of human diarrheagenic *Escherichia coli*. *J Clin Microbiol* 41(6)**,** 2669-2671. doi: 10.1128/jcm.41.6.2669-2671.2003.

van der Velden, A.W., Lindgren, S.W., Worley, M.J., and Heffron, F. (2000). *Salmonella* pathogenicity island 1-independent induction of apoptosis in infected macrophages by *Salmonella enterica* serotype typhimurium. *Infect Immun* 68(10)**,** 5702-5709. doi: 10.1128/iai.68.10.5702-5709.2000.

Vázquez-Boland, J.A., Kuhn, M., Berche, P., Chakraborty, T., Domínguez-Bernal, G., Goebel, W., et al. (2001). *Listeria* pathogenesis and molecular virulence determinants. *Clin Microbiol Rev* 14(3)**,** 584-640. doi: 10.1128/cmr.14.3.584-640.2001.

Xiao, L., Zhang, L., and Wang, H.H. (2012). Critical issues in detecting viable *Listeria monocytogenes* cells by real-time reverse transcriptase PCR. *Journal of food protection* 75(3)**,** 512-517. doi: 10.4315/0362-028x.jfp-11-346.

Zhang, W.-L., Bielaszewska, M., Liesegang, A., Tschäpe, H., Schmidt, H., Bitzan, M., et al. (2000). Molecular characteristics and epidemiological significance of shiga toxin-producing *Escherichia coli* O26 strains. *Journal of Clinical Microbiology* 38**,** 2134-2140.
