# Supplementary material for: Direct Metatranscriptome RNA-seq and Multiplex RT-PCR Amplicon Sequencing on Nanopore MinION – Promising Strategies for Multiplex Identification of Viable Pathogens in Food
Source: Front Microbiol. 2020 Apr 9;11:514. doi: 10.3389/fmicb.2020.00514 (PMC7160302; doi:10.3389/fmicb.2020.00514)
Supplement: Supplementary file 2 [file Data_Sheet_2.docx]

***Supplementary Image***

**Supplementary Figure 1. The growth curve of cocktail culture of *E. coli* O157:H7, *S. enteritidis* and *L. monocytogenes* at 0, 4, 8, 24 and 72 h in BHI and LJE at 37 ºC.** (A) The growth curve of cocktail culture of *E. coli* O157:H7 (3-log), *S. enteritidis* (3-log) and *L. monocytogenes* (6-log) at 0, 4, 8, 24 and 72 h in BHI. (B) The growth curve of cocktail culture of *E. coli* O157:H7 (3-log), *S. enteritidis* (3-log) and *L. monocytogenes* (6-log) at 0, 4, 8, 24 and 72 h in LJE. (C) The growth curve of cocktail culture of *E. coli* O157:H7 (3-log), *S. enteritidis* (3-log) and *L. monocytogenes* (4-log) at 0, 4, 8, 24 and 72 h in BHI. (D) The growth curve of cocktail culture of *E. coli* O157:H7 (3-log), *S. enteritidis* (3-log) and *L. monocytogenes* (4-log) at 0, 4, 8, 24 and 72 h in LJE.

(TIF)

**Supplementary Figure 2. The number of reads and read lengths of MinION R9.4 Rev D direct metatranscriptome RNA-seq and RT-PCR amplicon sequencing for BHI and LJE samples collected from 4 h and 24 h. Results of direct metatranscriptome RNA-seq.** (A) BHI-24 h; (B) LJE-24 h; (C) BHI-24 h with non-rRNA mapping; (D) LJE-24 h with non-rRNA mapping. Results of RT-PCR amplicon sequencing of (E) BHI-4 h; (F) LJE-4 h.

(TIF)

Supplementary Figure 3. Gel image of RT-PCR amplicons were used to verify the present of all three bacteria in the cocktail culture, and PCR was used to verify the complete removal of DNA contaminations using the protocol described above. (A) Non-multiplex RT-PCR of *E. coli* O157:H7 (*Ec*), *S. enteritidis* (*Se*) or *L. monocytogenes* (*Lm*) from 24-hour cocktail culture in LJE. From left to right: 1. 100-bp DNA Ladder (NEB); 2. *E. coli* O157:H7 in LJE 24-hour cocktail culture; 3. *S. enteritidis* in LJE 24-hour cocktail culture; 4. *L. monocytogenes* in LJE 24-hour cocktail culture. 5. Negative control (NC) of *E. coli* O157:H7 in LJE 24-hour cocktail culture; 6. Negative control of *S. enteritidis* in LJE 24-hour cocktail culture; 7. Negative control of *L. monocytogenes* in LJE 24-hour cocktail culture. (B) Multiplex RT-PCR (Multi) of *E. coli* O157:H7, *S. enteritidis* and *L. monocytogenes* 4-hour cocktail in LJE. From left to right: 1. 100-bp DNA Ladder; 2. Multiplex RT-PCR of *E. coli* O157:H7, *S. enteritidis* and *L. monocytogenes*; 3. Negative control of *E. coli* O157:H7 (PCR without RT); 4. Negative control of *S. enteritidis* (PCR without RT); 5. Negative control of *L. monocytogenes* (PCR without RT)*.*

(TIF)
