# Supplementary figures and images for: Direct Metatranscriptome RNA-seq and Multiplex RT-PCR Amplicon Sequencing on Nanopore MinION – Promising Strategies for Multiplex Identification of Viable Pathogens in Food
Source: Front Microbiol. 2020 Apr 9;11:514. doi: 10.3389/fmicb.2020.00514 (PMC7160302; doi:10.3389/fmicb.2020.00514)

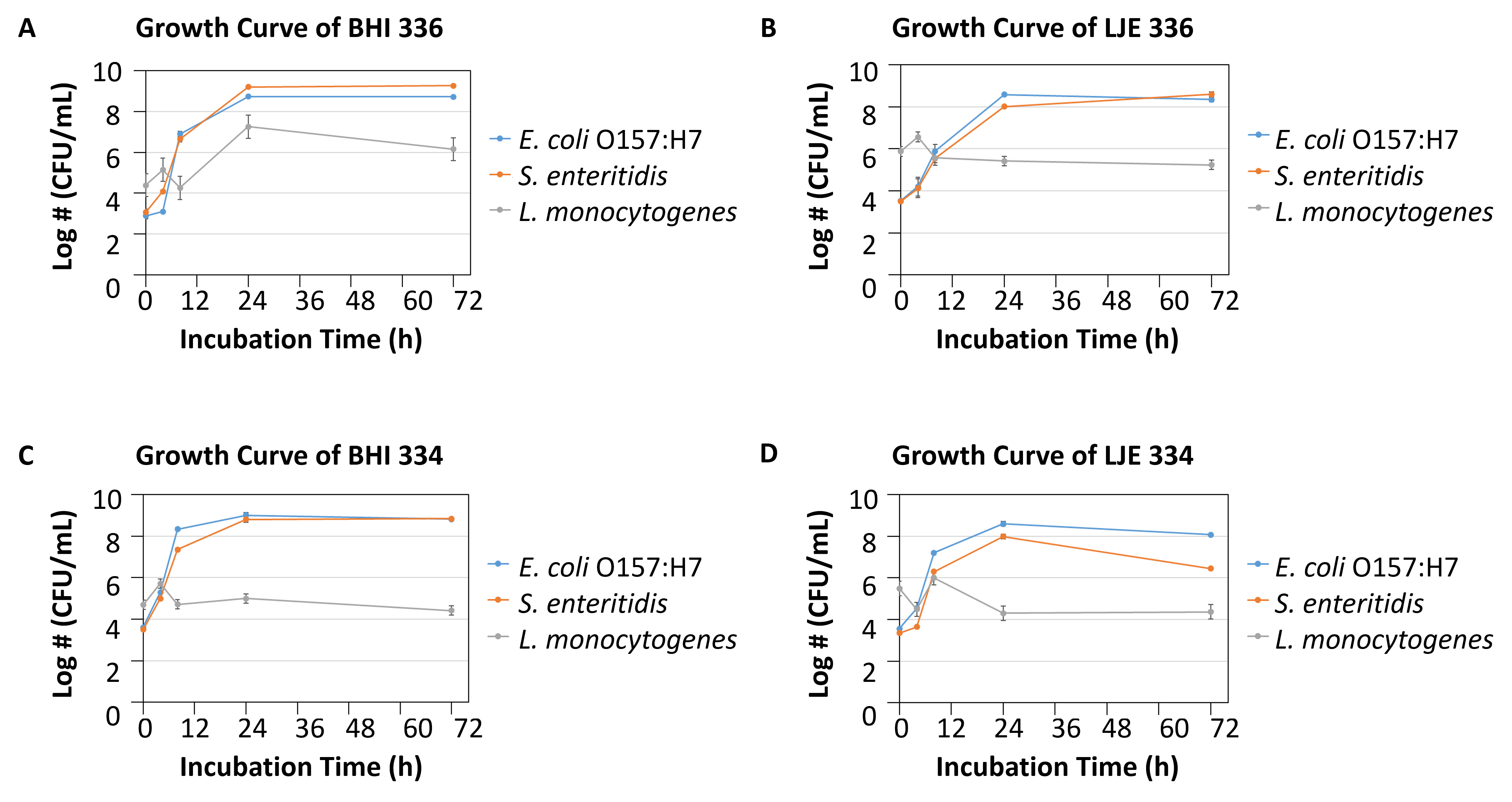

Supplement: Supplementary file 3 [file Image_1.TIF]

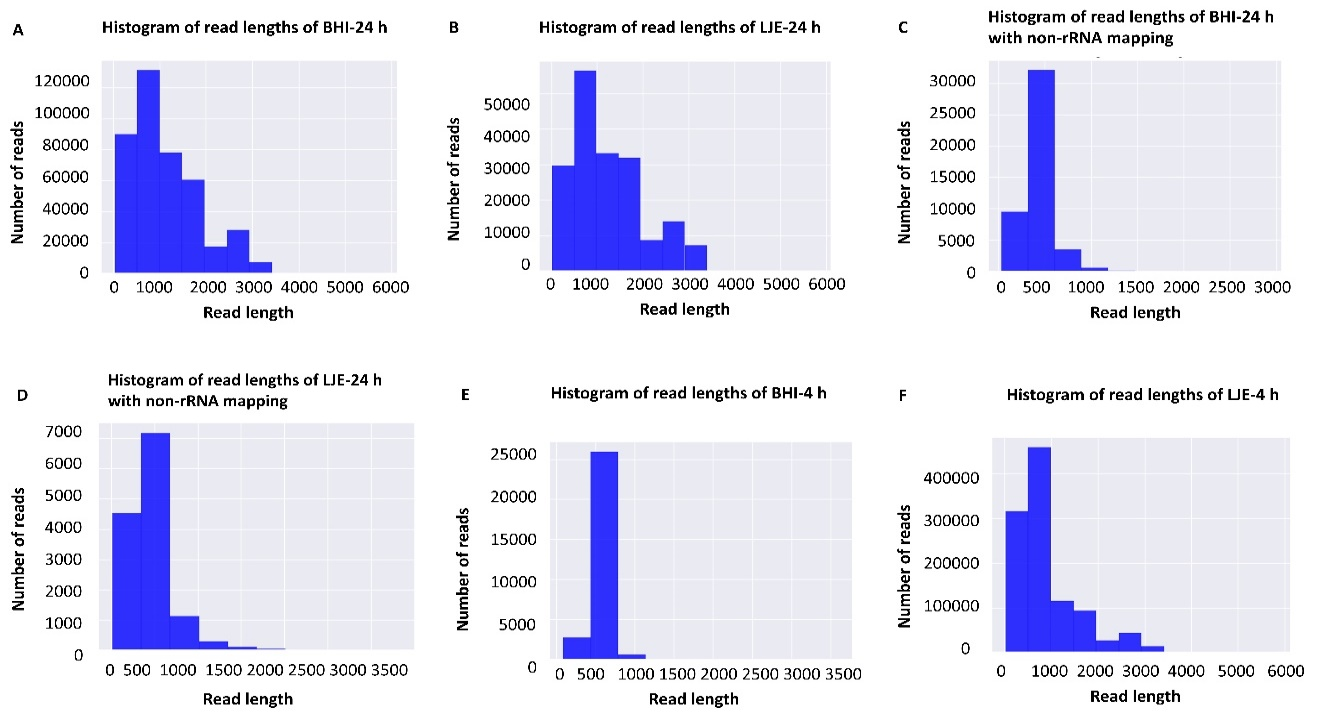

Supplement: Supplementary file 4 [file Image_2.TIF]

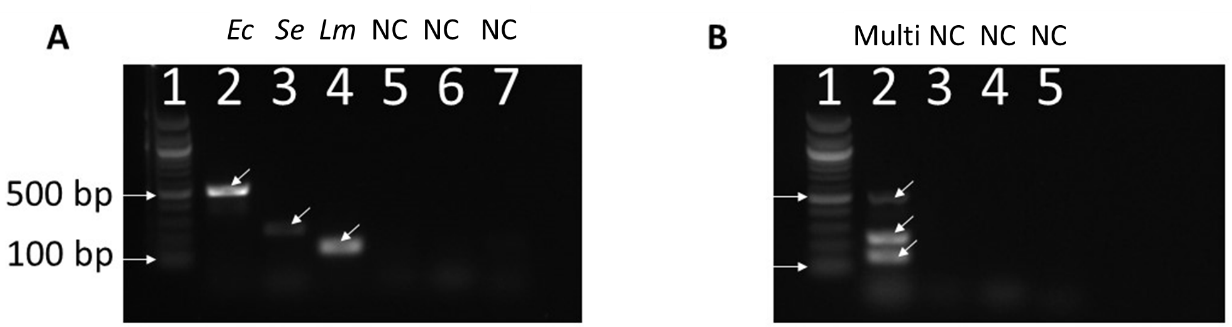

Supplement: Supplementary file 5 [file Image_3.TIF]
